# LC–MS Based Draft Map of the *Arabidopsis thaliana* Nuclear Proteome and Protein Import in Pattern Triggered Immunity

**DOI:** 10.3389/fpls.2021.744103

**Published:** 2021-11-08

**Authors:** Mohamed Ayash, Mohammad Abukhalaf, Domenika Thieme, Carsten Proksch, Mareike Heilmann, Martin Hartmut Schattat, Wolfgang Hoehenwarter

**Affiliations:** ^1^Department Biochemistry of Plant Interactions, Leibniz Institute of Plant Biochemistry, Halle, Germany; ^2^Institute for Biochemistry and Biotechnology, Martin-Luther University Halle-Wittenberg, Halle, Germany; ^3^Institute for Biology, Martin-Luther University Halle-Wittenberg, Halle, Germany

**Keywords:** proteomics, nucleus, *Arabidopsis*, protein trafficking, pattern triggered immunity

## Abstract

Despite its central role as the ark of genetic information and gene expression the plant nucleus is surprisingly understudied. We isolated nuclei from the *Arabidopsis thaliana* dark grown cell culture left untreated and treated with flg22 and nlp20, two elicitors of pattern triggered immunity (PTI) in plants, respectively. An liquid chromatography mass spectrometry (LC–MS) based discovery proteomics approach was used to measure the nuclear proteome fractions. An enrichment score based on the relative abundance of cytoplasmic, mitochondrial and Golgi markers in the nuclear protein fraction allowed us to curate the nuclear proteome producing high quality catalogs of around 3,000 nuclear proteins under untreated and both PTI conditions. The measurements also covered low abundant proteins including more than 100 transcription factors and transcriptional co-activators. We disclose a list of several hundred potentially dual targeted proteins including proteins not yet found before for further study. Protein import into the nucleus in plant immunity is known. Here we sought to gain a broader impression of this phenomenon employing our proteomics data and found 157 and 73 proteins to possibly be imported into the nucleus upon stimulus with flg22 and nlp20, respectively. Furthermore, the abundance of 93 proteins changed significantly in the nucleus following elicitation of immunity. These results suggest promiscuous ribosome assembly and a role of prohibitins and cytochrome C in the nucleus in PTI.

## Introduction

Subcellular compartmentalization is a defining characteristic of eukaryotic organisms and higher cell life. Cellular organelles are membrane enclosed spaces with specific architecture and physiological milieus. They are dynamic in nature undergoing morphological changes throughout the cell cycle and in response to environmental stimuli as well as moving throughout the hydrophilic cytoplasm and making and breaking contact with one another. The organelles contain specific sets of proteins mostly encoded in the nucleus and other biomolecules that facilitate their function in the cell. Extensive molecular traffic between the organelles co-ordinates and integrates their activity for complex biochemical and metabolic processes that may require diverse conditions and for optimal, overreaching cellular function.

The plant nucleus is among the larger cell organelles and encloses the genome, extensively reviewed by Petrovská et al. (2015) and Meier et al. (2017). It is encompassed by the nuclear envelope consisting of the inner (INM) and outer nuclear membranes (ONM) and traversed by nuclear pore complexes (NPCs). NPCs facilitate protein import into the nucleus and are the largest cellular multi-protein complexes comprising more than 30 nucleoporin proteins in *Arabidopsis thaliana* (Nups; Tamura et al., 2010; Tamura and Hara-Nishimura, 2013). The nuclear lamina is associated with residual parts of the NPCs and the INM (Aebi et al., 1986) and is composed of lamin-like proteins, most prominently of the NUCLEAR MATRIX CONSTITUENT PROTEIN 1 (NMCP1) family of which around 100 members have been characterized in more than 30 plant genomes (Ciska and Moreno Diaz de la Espina, 2013; Wang et al., 2013). The nuclear lamina is connected to the cytoskeleton *via* the LINC complex that is composed of SAD1/UNC84 (SUN) and KLARSICHT/ANC-1/SYNE-1 HOMOLOGY (KASH) proteins that in turn associate with INM and ONM and that expediates nuclear positioning and migration (Sosa et al., 2012; Zhou et al., 2012). The chromatin is anchored by the INM and is scaffolded on histone proteins, the mass of which is approximately equal to the mass of DNA (Wiśniewski et al., 2014). The cell cycle leads to changes in chromatin structure and chromosome positioning and indeed cell division leads to the complete breakdown of the nuclear envelope for the mitotic spindle to access the chromosomes (Kutay and Hetzer, 2008). Next to these facultative nuclear proteins that function in morphology, structure and replication of nucleus and DNA and are entirely location specific, the nuclear proteome is expanded by a host of proteins that are transiently imported upon demand. These are proteins that differentially regulate gene expression such as kinases and other components of signaling cascades that terminate in the nucleus and their substrates, DNA and chromatin associated proteins, transcriptional co-activators and repressors and transcription factors. Thus, the plant cell nucleus is populated by an estimate of several thousand proteins and the nuclear proteome is highly diverse dependent on the biological cell state.

In many cases proteins are the convergence points of organelle initiated second messenger or small molecule signaling, which imported into the nucleus, initiate a transcriptional response (Kmiecik et al., 2016). Both plastids and mitochondria generate reactive oxygen species (ROS) and Ca^2+^ fluxes that as organelle signals converge on specific kinases and transcription factors that convey the signal to the nucleus (Crawford et al., 2018). This type of retrograde signaling and sub-cellular protein trafficking between compartments has been shown to be instrumental in the cellular response to various types of abiotic stress as well as in the establishment of plant immunity.

Plant immunity is composed of multiple overlapping layers that show many of the same effects. The recognition of molecular patterns indicative of non-self (pathogen or microbe associated molecular patterns PAMPS or MAMPs) or of non-homeostasis self (damage associated molecular patterns DAMPs) by receptors is central to the initiation of defense. In the resistance to biotrophic pathogens, pathogens in the apoplast are first recognized by plasma membrane spanning pattern recognition receptors (PRRs) such as Leucine-rich repeat receptor like kinases (LRR-RLKs) or receptor like proteins (LRR-RPs) that recruit kinases to initiate signaling (Zhou and Zhang, 2020). Initial recognition and signaling is often potentiated by a host of other molecular recognition events that together propagate the signal (Zhang et al., 2020). The best studied example of these processes is signaling downstream of the LRR-RLK FLAGELLIN SENSING 2 (FLS2) that recognizes bacterial flagellin (Gómez-Gómez and Boller, 2000). Its 22 amino acid N-terminal epitope, flg22, is a commonly used elicitor that we have also used here (Meindl et al., 2000). The LRR-RP RLP23 is another closely related PRR that recognizes phytotoxic virulence factors ethylene-inducing peptide 1 (Nep1)-like proteins (NLPs) and initiates a similar response. Signaling is activated by perception of the characteristic 20 amino acids long peptide nlp20 (Albert et al., 2015) that we have also used in this study. Flg22 and nlp20 were investigated before in a transcriptomic study in *Arabidopsis* seedlings (Wan et al., 2019).

Early events following PAMP perception are the production of ROS and Ca^2+^ influx. Ca^2+^ is essential because it directly controls many immune regulatory proteins such as calcium dependent protein kinases (CPKs) and others (Boudsocq et al., 2010). Mitogen associated protein kinase (MAPK) signaling is another central pillar of immune signaling that leads to a broad range of events by way of their phosphorylated activated substrates (Bigeard et al., 2015). Many of these are transcription factors that orchestrate reprogramming of gene expression. Phytohormones, particularly salicylic acid (SA), jasmonic acid (JA), and ethylene (ET) also play important roles in regulating immunity (Pieterse et al., 2012). These events are collectively termed pattern triggered immunity (PTI) and shift the plant away from homeostasis to a state of immunity that is hallmarked by the production of pathogenesis-related (PR) proteins and antimicrobial secondary metabolites.

Research has shown that a growing number of proteins are located in two or more cellular organelles. Protein dual targeting has been reported among others for nucleus and mitochondria (Carrie et al., 2009), nucleus and plastids (Schwacke et al., 2007) and is most common for chloroplasts and mitochondria (Sharma et al., 2018). Protein dual targeting arose early in the evolution of land plants, at least 450 million years ago when *Physcomitrella patens* diverged from *A. thaliana* (Xu et al., 2013), is conserved among species and rarely lost once acquired (Carrie and Whelan, 2013). Dual targeted proteins generally have a primary function in only one of the targeted compartments. One hypothesis suggests that dual targeting may be a mechanism by which entire biochemical pathways can be copied and moved from one organelle to another (Martin, 2010). Proteins are targeted to their respective sub-cellular localizations by transport signals and for dual targeted proteins the respective gene has the coding capacity for multiple signals producing a protein with different transport signals or the signal peptide itself may be ambiguous giving rise to dual targeting (Peeters and Small, 2001). Alternatively a mature protein can be relocated from one organelle to another (Krause and Krupinska, 2009).

Liquid chromatography mass spectrometry (LC–MS) based proteomics is a powerful tool to investigate the entire protein complement of organelles. Thousands of proteins can be identified and quantified in a single experiment. The plant nucleus has not been extensively studied. Here we present a quantitative, high-quality draft analysis of the *A. thaliana* nuclear proteome including rearrangement of proteome architecture in PTI. We identified proteins that are present in the cytosol or other organelles or newly synthesized and then imported into the nucleus following PAMP exposure. Our dataset provides a list of potential candidate proteins for further study that may underlie, retrograde signaling and nuclear trafficking in the context of plant immunity. Bioinformatics tools were used to uncover proteins located (experimentally verified and predicted) in other organelles besides the nucleus. These are potential protein candidates for dual targeting that we provide for further investigation. Our work suggests these phenomena occur more frequently than previously reported.

## Materials and Methods

### Preparation of the Protoplasts

Thirty milliliters of 5days old *A. thaliana* cultured cells grown in the dark was centrifuged at 805 *g* for 5min at room temperature (RT). The pellets were resuspended in 30ml of 0.24M CaCl_2_. Then 15ml of this suspension, 20ml of 0.24M CaCl_2_ and 15ml of the enzyme solution (0.2% macerozyme, 0.67% cellulose, and 0.24M CaCl_2_) were transferred to a petri dish. The petri dish was incubated at RT overnight shaking at 45rpm. The content of the petri dish was centrifuged at 290 *g* for 5min at RT and the pellets were resuspended in 30ml of 0.24M CaCl_2_. The same centrifugation step was repeated but, the pellets were resuspended in 14ml of B5 sucrose solution (0.32% gamborg B5 medium, 1mg/L 2,4-D, and 0.28M sucrose at pH 5.5). The final suspension was centrifuged at 130 *g* for 5min at RT and was left for 5–10min on the rack. The floating protoplasts were collected from the top layer. Protoplast samples were supplemented with flg22 and nlp20, respectively, to a concentration of 1μM in solution and incubated for 16h at 18°C. Control samples were untreated and incubated similarly. These experiments were performed three times independently (three biological replicates for each condition).

### Preparation of Nuclear and Cellular Fractions

Four milliliters of protoplasts were mixed with 9ml of NIBA [25% v/v NIB 4x (nuclei isolation buffer), 1mM DTT and 1% protease inhibitor] in a falcon tube and kept on ice for 10min. Triton X-100 was added to an in solution concentration of 0.1% and the suspension was gently mixed for 5min. Three consecutive centrifugation steps were done each at 1,000 *g* for 15min at 4°C. After the first two steps the pellets were resuspended in 4ml NIBA containing 0.1% triton X-100. The supernatants were retained as the cellular fraction. Then the pellets were resuspended in 4ml NIBA (washing step) and centrifuged as before. After the third step, the pellets were resuspended in 300μl extraction buffer and transferred to a 1.5ml Eppendorf tube (nuclear fraction, NF). NIBA 4x and extraction buffer were taken from a commercially available nuclear isolation kit (CELLYTPN1-1KT for plants, SIGMA).

### Staining and Microscopy

A fluorescence microscope (Axioplan2 imaging, Carl Zeiss) with a DAPI filter was used to visualize DAPI stained nuclei. One hundred microliter of 5μg/ml DAPI were added to 10μl of the nuclear fraction and kept in darkness for 15min. Then, 10μl of this solution were used for microscopy.

### Extraction of Nuclear Proteins

Two hundred microliter of extraction buffer (containing 1% protease inhibitor cocktail, Sigma P9599) were added to the NF. The sample was vortexed at 1,800rpm for 30min at RT and then it was sonicated in an ultrasonicator for 10min. Then, the sample was centrifuged in a fixed rotor angle centrifuge at 12,000 *g* for 10min at RT. The supernatant was collected representing nuclear proteins.

### Extraction of Cellular Proteins

Five milliliter of *CF* was mixed with 45ml of 100mM ammonium acetate in methanol. The mixture was kept at −20°C overnight and then three centrifugation steps were done with a swinging bucket rotor centrifuge at 3,200 *g* for 15min at 4°C. The pellets from the first two centrifugation steps were washed with 3ml of 20% 50mM ammonium bicarbonate and 80% acetone and the final pellets were left to dry at RT. The dried pellets were solubilized in urea buffer (8M urea and 50mM Tris) and constituted cellular proteins.

### Western Blot Analysis

Five microgram of the protein extracts were separated into one gradient SDS-PAGE (20–4%, Serva). The proteins were transferred to a nitrocellulose membrane using a wet blot technique (Protran, GE Healthcare). The membranes were blocked with 3% fat-free dry milk (BioRad) in TBS (50mM Tris–HCl pH7.5, 150mM NaCl). The blocked membranes were incubated with polyclonal anti-histone H3 antibody (Agrisera) and a secondary anti-rabbit antibody coupled to HRP. Detection was performed with SuperSignal™ West Femto Maximum Sensitivity Substrate (Thermo) and the signal was recorded with the Fusion Solo S Chemiluminescence Imaging System (VWR) using a 16-bit CCD camera.

### In-Solution Digestion of Proteins Using Trypsin

The protein samples were reduced by addition of DTT solution (29.9μg/μl). Then, the samples were kept at 22°C for 1h shaking at 450rpm. Samples were alkylated by the addition of iodoacetamide solution (35.9μg/μl) and kept at 22°C for 1h shaking at 450rpm in darkness. Again, the reducing solution was added to samples and was kept at 22°C for 1h with shaking. Fifty millimolar ammonium bicarbonate pH 8.5 was added to each sample. Trypsin (0.2μg/μl) was added to a ratio of 1:50. Protein digestion was allowed to proceed overnight at 37°C shaking at 750rpm. The next day, samples were dried in a vacuum concentrator.

### Stage-Tip C18 Peptide Desalting (Stop-and-Go Extraction)

Dried peptides were dissolved in 200μl 0.1% formic acid (FA). Desalting was done using in house produced C18-STAGE-Tips. STAGE-Tips were inserted into 1.5ml Eppendorf tubes and conditioned with 100μl 80% acetonitrile (ACN), 0.1% FA by centrifugation at 1,500 *g* for 2min at RT. Then, they were equilibrated two times with 100μl 0.1% FA with subsequent centrifugation at 1,500 *g* for 2min at RT. The dissolved peptides were applied to STAGE-Tips and centrifuged twice at 1,500 *g* for 2min at RT. The flow-throughs were discarded. The STAGE-Tips were washed twice with 100μl 0.1% FA and centrifuged as before. The flow-throughs were discarded. STAGE-Tips were inserted into new 1.5ml Eppendorf tubes and elution was done twice by adding 50μl of 80% ACN, 0.1% FA followed by centrifugation at 1,500 *g* for 1min at RT. The eluates (peptides) were combined and dried in a vacuum concentrator.

### Liquid Chromatography and Mass Spectrometry

The dried peptides were dissolved in 10μl of 5% ACN, 0.1% TFA. The samples were analyzed on a Q Exactive Plus mass spectrometer equipped with an EASY nanoLC-1,000 liquid chromatography system (both from ThermoFisher Scientific). A flow rate of 250nl/min was used. Peptides were separated using an analytical column ES803A (ThermoFisher) and a gradient increasing from 5 to 40% of solvent B (ACN in 0.1% FA) in 540min followed by 13min of isocratic flow at 80% of solvent B (for cellular proteins). On the other hand, the nuclear proteins peptide samples were separated using a gradient inclining from 5 to 35% of solvent B (ACN in 0.1% FA) in 450min followed by 20min of incline to 80% solvent B and finally fixed at 80% solvent B for 70min. The spray voltage was set to 1.9 KV and the capillary temperature to 275°C.

A Data-Dependent Acquisition (DDA) scan strategy was used, where one MS full scan was followed by up to 10 MS2 scans of product ions from the 10 most abundant precursor ions. The MS full scan parameters were: AGC target 3E+06, resolution 70,000 and max injection time (IT) 100ms. The MS2 parameters were: resolution 17,500, Max IT 50ms, dynamic exclusion duration 40s, ACG target 5E+04 and isolation window 1.6m/z.

### Identification and Quantification of Peptides and Proteins

Peptide and by inference protein identification was done by matching the MS raw data with *in silico* generated peptide ion *m/z* and MS2 spectral peak lists. The TAIR10 protein database (14,486,974 residues, 35,394 sequences) was searched using the Mascot search engine V2.5.1 coupled to the Proteome Discoverer 2.1.1.21 (Thermo Fisher Scientific). The enzyme specificity was set to trypsin with tolerance of two missed cleavages. Ion m/z error tolerance was set to 5ppm and 0.02Da for precursor and fragment ions, respectively. Carbamidomethylation of cysteine was set as a static modification and oxidation of methionine as a variable modification. Peptide spectral match (PSM), peptide and protein level false discovery rates (FDR) were determined by a decoy database search. A significance threshold of *α*=0.01 was used for PSM and peptide level identifications. For the protein level: *α* of 0.05 was tolerated. The PSM count was used as protein abundance quantitative index (PQI). Protein grouping was inferred based on the principal of parsimony and only master proteins (protein group member that best explains the set of peptides used for inference) were retained. In the case of duplicate gene models producing individual master proteins, the first gene model was retained (this was the case in less than 1% of master proteins).

### Bioinformatics Data Analysis

Gene ontology analysis of the curated nuclear proteome was performed using the DAVID Bioinformatics resources 6.8 (Huang et al., 2009a,b) using default parameters. *Arabidopsis thaliana* was used as background and TAIR_ID was used as identifier. Functional annotation chart was created with threshold of count: two and ease: 0.1. Proteins annotated to the nucleus, with GOTERM_CC_DIRECT, were further clustered using high classification stringency. Subcellular location of the curated nuclear proteome was checked with SUBA4 (Hooper et al., 2017) using experimental locations inferred by fluorescent protein (FP) or MS/MS studies (retrieval from SUBA4 was done in January 2020 and rechecked in February 2021). LOCALIZER 1.0.4 (Sperschneider et al., 2017) was used to predict organelle subcellular localization by searching for targeting sequences such as NLS in protein primary structure and by predicting transit peptides. To further evaluate the possible biological role of the proteins in significant functional categories, their AGI codes were used to query the STRING database (Szklarczyk et al., 2019) for physical interaction setting the stringency to highest confidence interactions, which we have shown to be true positive previously (Hoehenwarter et al., 2013), using experiments, databases, co-occurrence and co-expression as interaction sources and showing only interactions between proteins in the input set. All raw and metadata have been deposited to ProteomeXchange Consortium *via* the Pride partner repository with the dataset identifier PXD024349.

### Collective Data Analysis

Mean PSM value of each protein in all measurements of the nuclear fraction (μNp_n_) and the cellular fraction (μCp_n_) were calculated. These values were used to formulate two scores, the nuclear and cellular enrichment scores (Npf_n_ and Cpf_n_) that express the ratio of the abundance of protein (*n*) in the nuclear and the cellular fraction, respectively, (Equations 1 and 2).


$${\mathrm{Npf}}_{n}=\frac{\mu Np_{n}}{\mu Np_{n}+\mu Cp_{n}}$$ (1)

$$\mathrm{Cpfn}=\frac{\mu Cp_{n}}{\mu Cp_{n}+\mu Np_{n}}$$ (2)

### Statistical Data Analysis

The matrix of curated nuclear proteins PQI (PSM) values of all samples was imported to Perseus software v.1.6.6.0 (Tyanova et al., 2016). The PQI values were grouped into three groups (control, flg22, and nlp20). Proteins that did not have a value in at least five of the six measurements of at least one group were discarded. The individual measurements (columns) were unit vectors normalized. Multiple sample test (ANOVA) was performed for the three groups in order to assess the significance of changes in abundance between conditions using permutation-based FDR multiples testing correction with an FDR significance threshold *α* of 0.05 and 250 permutations. *Post hoc* test (FDR=0.05) was performed to identify the significant group pairs. Proteins with statistically significant changes in their abundance were kept and their values *Z*-score transformed. Hierarchical clustering was performed using Pearson correlation as distance measure for row clustering and Spearman correlation for columns.

## Results

In this study we set out to produce a high-quality draft catalog of the *A. thaliana* nuclear proteome based on isolation of nuclei and mass spectrometric (MS) measurement of nuclear protein fractions. Beyond this we investigated the quantitative changes in protein abundance in the nuclear proteome in the three biological scenarios (control, flg22, and nlp20). To do this we chose to expose *Arabidopsis* protoplasts released from cells in culture by enzymatic digestion of cell walls to the elicitors. Additionally, we took first steps towards identifying candidate proteins imported into the nucleus in two related forms of PTI elicited using flg22 and nlp20, respectively, on a large scale.

**Figure 1:**
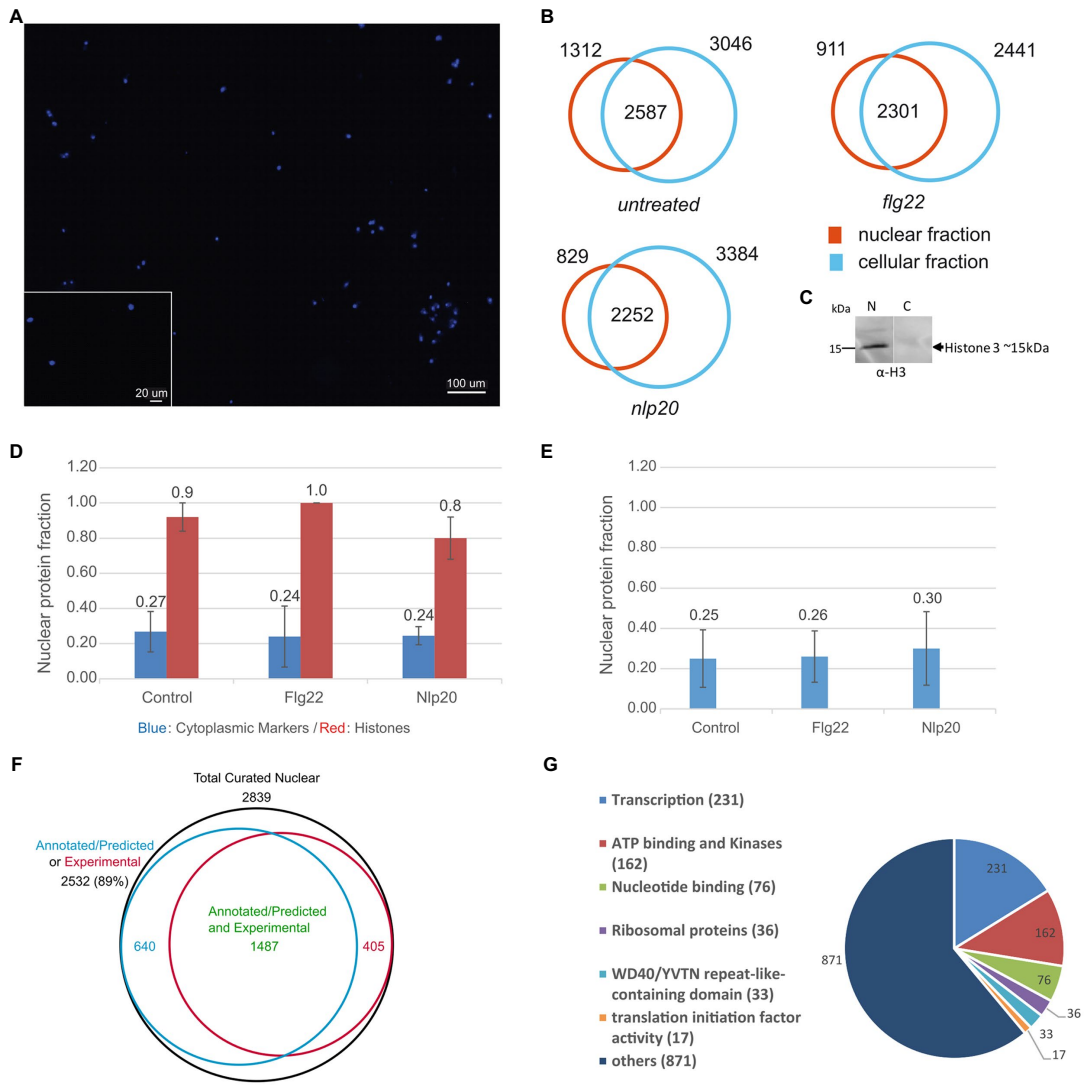
Extraction of the nuclear proteome. **(A)** DAPI staining fluorescence microscopy of nuclei isolated from dark grown *Arabidopsis thaliana* cell culture. **(B)** Total proteins identified in nuclear and cellular fractions in three independent experiments in untreated, flg22 and nlp20 treated cell cultures. **(C)** Western blot of nuclear (N) and cellular (C) proteins with anti-Histone H3 antibody, respective Mw range is shown. **(D)** Median nuclear enrichment scores (Npfn values) in three independent experiments of all identified histone proteins and of cytoplasmic markers (two cytoplasmic markers were absent in shared proteins in flg22 and nlp20 conditions and were not used in both conditions). Error bars denote median absolute deviation. **(E)** Median Npfn values of mitochondrial and Golgi markers. Error bars denote median absolute deviation. **(F)** VENN diagram showing sets of experimentally validated or annotated/predicted nuclear proteins, comprising our data set. **(G)** DAVID gene ontology classification of 1,426 proteins of the untreated nuclear protein fraction annotated as nuclear proteins by DAVID bioinformatics tool.

In order to specifically gain access to the nuclear proteome, nuclei were isolated from the protoplast incubated at 18°C for 16h under three conditions: 1μM flg22, 1μM nlp20 and untreated in case of control. This was repeated twice for a total of three independent experiments. The cellular suspension resulting as a product of the isolation procedure was also retained and used to prepare the cellular protein fraction comprising all proteins with the exception of those in the nucleus. The isolated nuclei were characterized by fluorescence microscopy after DAPI staining which attested to their successful isolation in an intact and round form (Figure 1A). Nuclear proteins were extracted, and in-solution digested with trypsin along with the cellular proteins. The dissolved peptides were analyzed with LC–MS using a Data-Dependent Acquisition (DDA) scan strategy. In all three experiments, the MS analysis identified 3,899, 3,212, and 3,081 protein groups in total (set of proteins identified with a non-redundant peptide set, hence referred to as proteins) in the nuclear fraction of untreated, flg22 treated and nlp20 treated samples, respectively. Likewise, 5,633, 4,742, and 5,636 proteins were identified in the cellular fractions of the respective samples (Supplementary File 1- Tables 1–3 and Tables 7–9; Supplementary File 2- Tables 1–6). The overlap between the fractions was 2,587, 2,301, and 2,252 proteins, respectively, (Figure 1B).

### Defining the *Arabidopsis thaliana* Nuclear Proteome

A central issue in all organelle isolation procedures is the purity and integrity of the preparation. Conventionally the purity of the extracted nuclear proteome is assessed by western blot of nuclear protein markers, often histones. We also did this using antibody against histone H3 (Figure 1C) but in addition devised an approach to assess quality directly from the MS data. We calculated the fraction of each protein’s abundance in the nuclear and cellular protein fractions independently by way of the acquired MS data as mentioned in the methods section under collective data analysis. This can be interpreted as an enrichment score which in brief is the ratio of a protein’s MS signal in the nuclear or cellular fraction to its total MS signal in both fractions. Thus exclusive detection in the nuclear fraction would give a nuclear enrichment score (Npf_n_) of one whereas the score would be 0 if it were detected only in measurements of the cellular fraction. The score for the cellular fraction (Cpf_n_) would be the inverse.

The median nuclear enrichment score (Npf_n_) of all histones in the control, flg22 and nlp20 samples is shown in Figure 1D (for each histone see Supplementary File 1- Tables 4–6). All samples show enrichment of histones indicating successful isolation of nuclei and extraction of the nuclear proteome. In addition, Npfn values of SUN2, KAKU4, WIP3, WIT1, and MAD1/NES1, five NE/INM proteins, were equal to 1 (exclusive presence in the nuclear protein fraction) indicating nuclei were isolated with the NE largely intact. To get an impression of the extent of inevitable contamination of the experimental nuclear proteome by cellular proteins, the fraction of the abundance of nine *bona fide* cytoplasmic markers (phosphoenolpyruvate carboxylase 1, phosphoenolpyruvate carboxylase 2, phosphoenolpyruvate carboxylase 3, Actin 1–3, Actin 7, Actin 8, Actin 12, sucrose phosphate synthase 1F and sucrose phosphate synthase 2F) were used. In contrast to histones, the median nuclear enrichment score (Npf_n_) of these markers was low (Figure 1D). In addition, we calculated median Npf_n_ values for the known mitochondrial and golgi apparatus markers, voltage dependent anion channel 1, 2, and 3 as well as isocitrate dehydrogenase 1 and subunit 2 (mitochondrion) and coatomer gamma-2 subunit (golgi apparatus) which also were in the range of the cytoplasmic markers (Figure 1E). FD-GOGAT and FNR1 and 2 which are known plastid markers were completely absent from nuclear fractions. Together these results show that the isolation of nuclei was successful and of high purity.

Regarding the nuclear proteome, the proteins shared by both nuclear and cellular fractions (Figure 1B, intersections) may indeed be common to both and underlie some type of trafficking between nucleus and other organelles or cytoplasm or may simply be inevitable experimental contaminations of the nuclear fraction. To address this issue, we decided to use the median Npf_n_ values of the cytoplasmic markers (0.27, 0.24, and 0.24 respectively) described above which are known not be present in the nucleus, as arbitrary cut off limits to define contamination in the three biological conditions and produce a curated set of nuclear proteins. All proteins with Npf_n_ values higher than this cut off limit were considered as genuinely localized in the nucleus and thus as nuclear proteins under the applied experimental conditions whereas those proteins with Npf_n_ values lower than the cut off limit were considered experimentally produced cellular protein contaminants and were discarded. The used cytoplasmic markers were discarded. This led to a curated set of nuclear proteins consisting of 2,839, 2,259, and 2096 proteins under control, flg22 and nlp20 conditions (Supplementary File 1- Tables 4–6).

To further validate the nuclear proteomes, we analyzed the curated protein lists with the DAVID Bioinformatics resources 6.8 gene ontology tool (Huang et al., 2009a,b) and LOCALIZER (Sperschneider et al., 2017), a software that predicts organelle subcellular localization by searching for targeting sequences such as NLS in protein primary structure. We also compared our experimentally determined proteomes with previously published nuclear/sub-nuclear proteomes and nuclear localized proteins by FP (Bae et al., 2003; Calikowski et al., 2003; Pendle et al., 2005; Sakamoto and Takagi, 2013; Bigeard et al., 2014; Chaki et al., 2015; Palm et al., 2016; Hooper et al., 2017; Goto et al., 2019; Mair et al., 2019; Tang et al., 2020). As a result, 89% of the nuclear proteins in each condition consisted of either experimentally verified nuclear proteins or proteins annotated/predicted to be localized in the nucleus (redundancy was removed), (Supplementary File 1- Tables 4–6; Figure 1F). This underscores the high quality of our nuclear proteome preparation.

The DAVID bioinformatics tool was used to further annotate the nuclear proteins and classify them according to their function. One thousand four hundred twenty-six proteins of the proteome measured under untreated conditions were classified initially as belonging to the nucleus with a Benjamini corrected value of *p* of 1.3E-44. This set was then further input into DAVID. These proteins were categorized into 83 clusters and six main protein classes (Figure 1G; Supplementary File 1- Table 10). The six main classes were: transcription (231 protein), ATP binding and kinases (162 proteins), nucleotide binding (76 proteins), ribosomal proteins (36 proteins), WD40 (33 proteins) and translation initiation factor activity (17 proteins).

Proteins annotated as related to the process of transcription, i.e., transcription factors (TFs) and transcriptional co-activators were classified into families, as shown in Supplementary File 1- Table 11, a total of 258 in all three conditions; control and elicited. The top three transcription factors families pertaining to the number of proteins identified were bZIP (13 proteins), WRKY (eight proteins) and Trihelix (eight proteins). According to Riaño-Pachón et al. (2007) there are a total of 2,304 TFs and regulators in *A. thaliana*. We checked the number of proteins annotated to transcription identified in a recent paper addressing the *Arabidopsis* nuclear proteome (again using DAVID) that employed the bioID strategy for specific identification of nuclear proteins (Mair et al., 2019). It was 266, similar to our results. Furthermore, we checked the number of TFs in our data and in concatenated data from a number of previously published nuclear proteomics studies (Bae et al., 2003; Calikowski et al., 2003; Pendle et al., 2005; Sakamoto and Takagi, 2013; Bigeard et al., 2014; Chaki et al., 2015; Palm et al., 2016; Goto et al., 2019; Mair et al., 2019; Tang et al., 2020) that are found in PlantTFDB (Jin et al., 2016; Tian et al., 2019), a database of plant transcription factors. One hundred thirty-eight TFs were identified in our data set whereas 231 were identified in the data from the other studies. The overlap was 84 TFs. Finally, we checked the number of proteins annotated to transcription (by DAVID) identified in the cellular fractions we isolated from all three conditions amounting to only six. Thus, this meta-analysis of TFs identified in our and other *Arabidopsis* nuclear proteomic studies shows that our LC–MS measurements of the nuclear protein fraction allowed deep insight into the transcription factor landscape in the nucleus which attests to the sequencing depth of our method.

Nuclear envelope proteins were also identified in our study. Forty-four proteins were reported to be localized in the nuclear envelope, inner and outer membrane based on FP experimental evidence in SUBA4. Eighteen of these (41%) were identified in our study in the nuclear fraction. Six hundred ninety-two proteins were found in SUBA4 with the same localization based on FP and MS experimental evidence, our study identified 391 of these (57%). All together our results constitute a high-quality catalog of the nuclear proteome of *A. thaliana* cell culture under homeostasis and induced immunity.

### LC–MS Based Candidate List of Putative Dual Targeted Proteins

To identify proteins that potentially underlie dual targeting to the nucleus and more than one organelle, we analyzed our curated nuclear protein list under control conditions with SUBA4 (Hooper et al., 2017), to check for proteins identified in previous experiments in plastids and mitochondria using FP or MS/MS. Four hundred eighty-four proteins in our nuclear list were also found to be experimentally localized in the plastid and 477 in the mitochondrion, indicating their possible dual targeting to the nucleus and these two organelles. Localization in plastid and mitochondrion of 67 (14%) and 71 (15%) of these was based on FP analysis meaning confirmation of sub-cellular localization by an orthogonal method. Out of the 484 putative dual- targeted plastid proteins, 268 proteins (55%) contained NLS, which were further refined to a set of 84 proteins containing both NLS and a predicted chloroplast transit peptide. In contrast only 37% of all proteins contained in SUBA4 experimentally shown to be localized in the plastid also had NLS, a substantially lower percentile. Out of 477 putative dual targeted mitochondrial proteins, 244 proteins contained NLS (51%) and again 84 showed both NLS and predicted mitochondrion transit peptide (Table 1; Supplementary File 1- Tables 15 and 16). Only 31% of all mitochondrial proteins in SUBA4 also had the NLS, again a substantially lower fraction than in our experimental set, underscoring possible dual targeting. We compared our lists of dual targeted candidate proteins with previously published nuclear and sub-nuclear proteomes (as mentioned before) and analyzed them with SUBA4 to determine experimental localization in the nucleus. We found 57 and 76 of our potentially dual targeting candidate proteins (nucleus-plastid and nucleus-mitochondrion, respectively) were to our knowledge not reported in the nucleus before. All of the proteins in these two sets contained NLS. Thirty-three (nucleus-plastid) and 36 (nucleus-mitochondrion) contained both NLS and respective predicted transit peptides (Table 1; Supplementary File 1- Tables 15 and 16).

**Table 1:** Putative dual targeted proteins (found in both organelles).

Nucleus – plastid		Nucleus – mitochondrion	
Curated nuclear proteome (control)	2,839	Curated nuclear proteome (control)	2,839
Proteins with alternative sub-cellular location (SUBA4)	484	Proteins with alternative sub-cellular location (SUBA4)	477
Containing NLS	268	Containing NLS	244
Containing NLS and predicted chloroplast transit peptide	84	Containing NLS and predicted mitochondrion transit peptide	84
Newly identified containing NLS	57	Newly identified containing NLS	76
Newly Identified containing NLS and predicted transit peptide	33	Newly Identified containing NLS and predicted transit peptide	36

### Protein Import Into the Nucleus Under flg22 and nlp20 Stimulus

**Figure 2:**
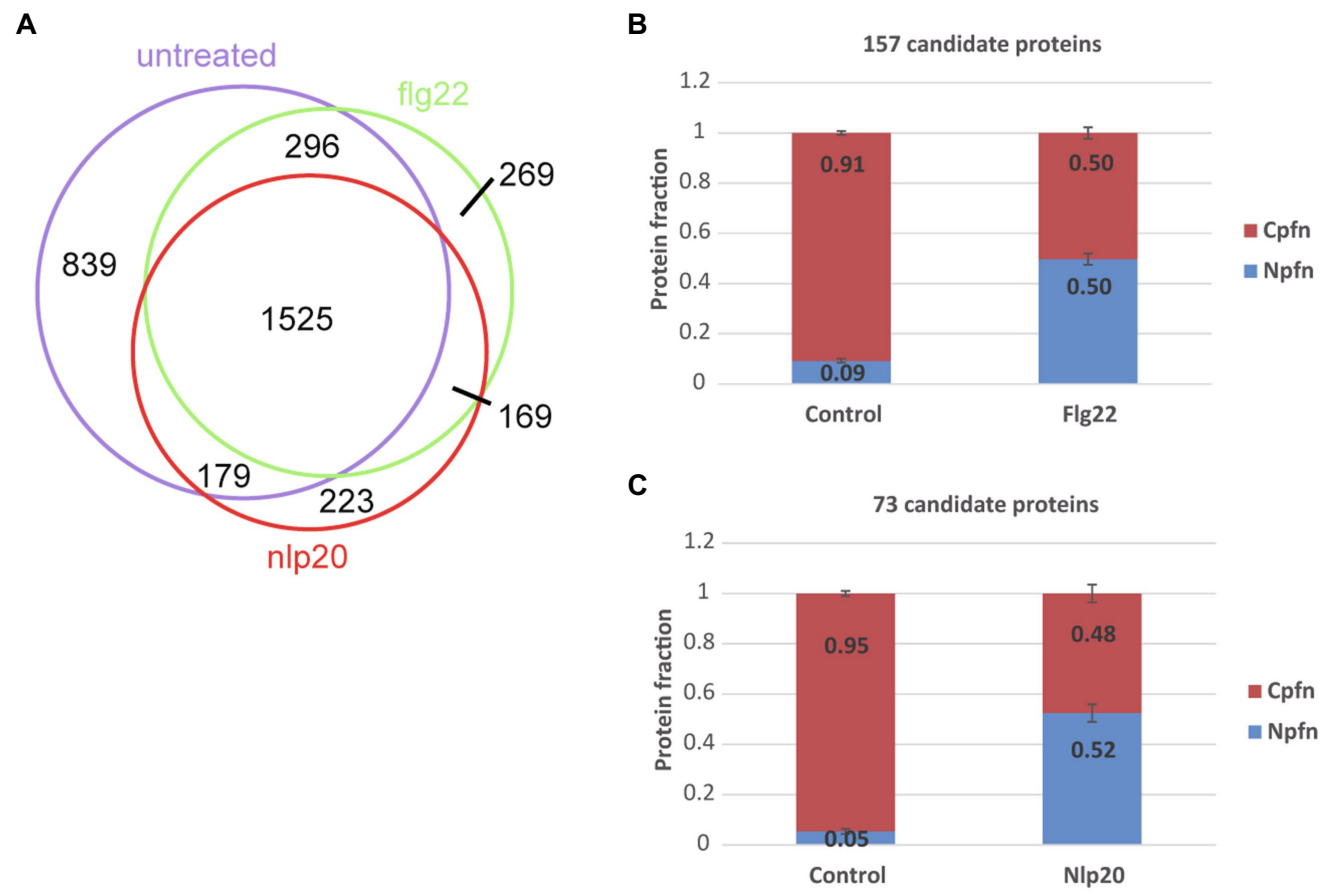
Protein import into the nucleus following elicitation of pattern triggered immunity (PTI). **(A)** Intersections of curated nuclear protein fractions extracted from untreated and flg22 and nlp20 treated protoplasts. **(B)** Mean nuclear and cellular protein enrichment scores (Npfn and Cpfn values) of 157 proteins identified in both the nuclear and cellular protein fractions without and following flg22 treatment. Error bars denote standard error. **(C)** Mean nuclear and cellular protein enrichment scores (Npfn and Cpfn values) of 73 proteins identified in both nuclear and cellular protein fractions without and following nlp20 treatment. Error bars denote SE.

We compared the curated nuclear proteins identified under control, flg22 and nlp20 conditions as shown in Figure 2A. One thousand five hundred twenty-five proteins were common to all three conditions, 269 and 223 were specific to flg22 and nlp20, respectively. This means that in our experiments considering our detection limits, these proteins appeared in the nucleus after elicitation of immunity with one of the two elicitors. These proteins could originate from the cytosol or other organelles and be imported upon PAMP perception or newly synthesized and then imported. In order to investigate this further, we first checked the cellular proteins measured under the non-elicited control conditions for the presence of these specific proteins. One hundred fifty-seven out of the 269 proteins appearing in the nucleus after flg22 exposure and 73 out of the 223 proteins appearing after nlp20 exposure were also measured in the cellular fraction without elicitation. Secondly, the nuclear enrichment (Npf_n_) and cellular enrichment scores (Cpf_n_) of the proteins were compared between control and flg22 and nlp20 elicited samples for the two sets of putatively imported proteins (157 and 73 proteins respectively). The Cpf_n_ decreased in both sets upon induction of immunity with either flg22 or nlp20 when compared to untreated samples. The Npf_n_ increased proportionally in elicited samples when compared to control (Figures 2B,C). The almost perfect proportionality and unitary sum is an indicator that these sets of candidate proteins were trafficked to the nucleus from the cytosol or some other cellular organelle upon elicitation of immunity wherein they could play some function. Both sets of proteins are shown in Supplementary File 1- Table 12.

We were interested in the re-localization of proteins from mitochondrion to the nucleus. To investigate this further, we first checked the 157 putatively imported proteins for the presence of predicted mitochondrion transit peptides by Localizer and we found 18 proteins with transit peptide predictions. Secondly, the MS raw data was researched with no enzyme specificity to identify peptides with non-tryptic N-termini generated by *in vivo* cleavage. If these non-tryptic cleavages demarcate the protein’s N-terminus and match transit peptide cleavage sites, then it suggests transit peptide cleavage of the protein *in vivo*. As shown in Table 2, the initial N-terminal part of primary structure of two proteins contain identified peptides with non-tryptic N-terminal cleavage sites (no R or K before, F was found in both), indicating the peptide sequences preceding the identified peptides (1–30 in first protein and 1–25 in second) were not cleaved by trypsin. This could imply that the two proteins were identified in the nucleus in an already cleaved form without the peptide sequences 1–30 and 1–25, respectively. These two proteins are known mitochondrial proteins, the first one contains a transit peptide at position 1–30 as investigated before (Carrie et al., 2015) and the second has a transit peptide at position 1–24, predicted by Localizer. This may suggest that these two proteins may be re-localized to the nucleus in their cleaved forms. This type of analysis provides putative candidates which need further verification by orthogonal methods.

**Table 2:** Re-localization of mitochondrial proteins to the nucleus.

Name	Initial part of protein sequence			Transit peptide
Fifty-one kilodalton subunit of complex I (AT5G08530)	1	30↓	52	1–30
	**MAPVRGILGLQRAVSIWKESNRLTPALRS** **F**STQAASTSTTPQPPPPPPPPEK			
NAD-dependent malic enzyme 2 (AT4G00570)	1	24↓	37	1–24
	**MMWKNIAGLS KAAAAARTHGSRRC** FSTAIPGPCIVHK			

Identified peptides are underlined. Transit peptides are in green. Arrow denotes the non-tryptic cleavage site.

### Comparison of Nuclear Proteomes Under flg22 and nlp20 Stimuli

**Figure 3:**
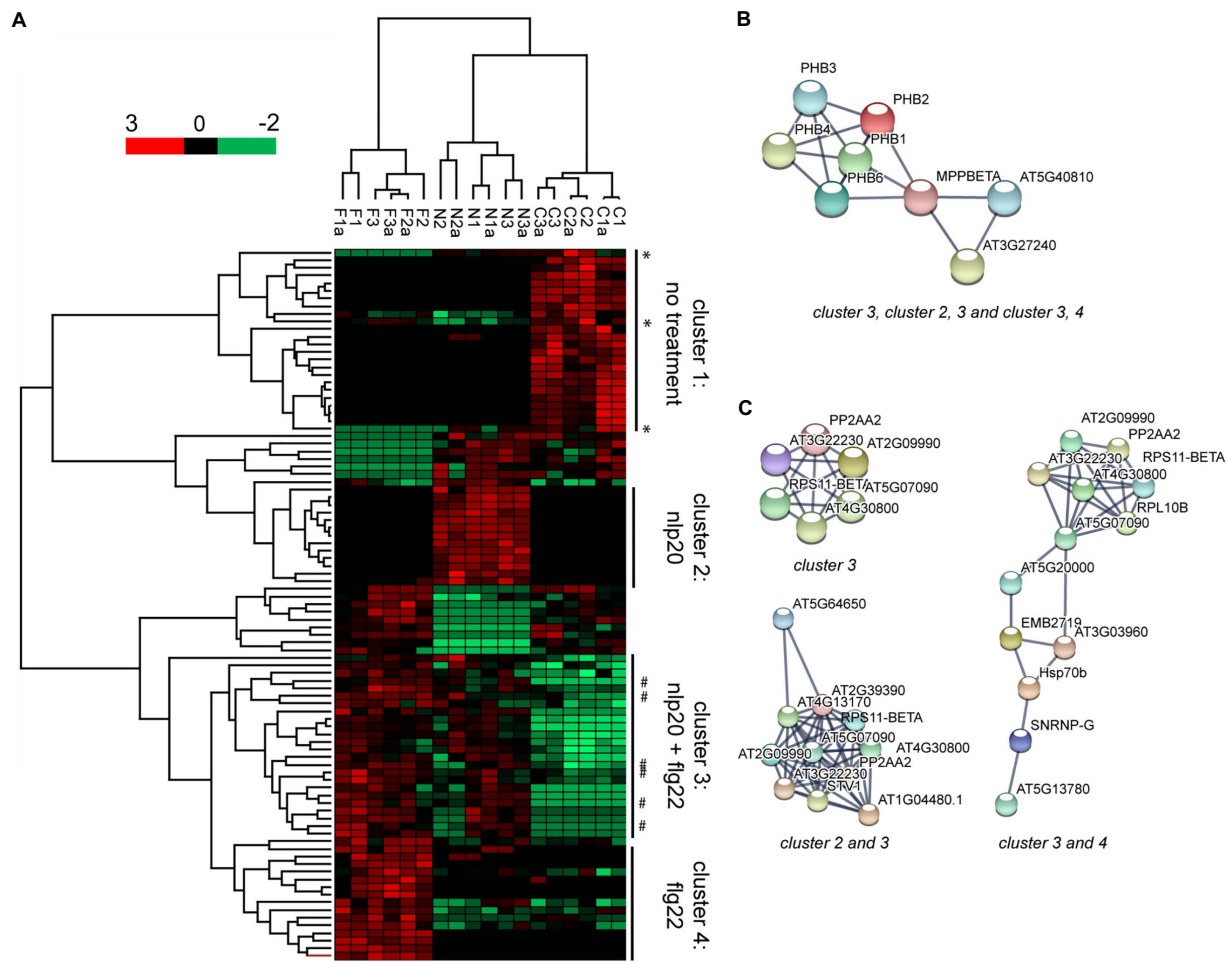
Ninety-three proteins showing significant changes in their abundance in the nuclear protein fraction following flg22 or nlp20 treatment. Multiple sample significance testing (ANOVA) was done, with false discovery rates (FDR) multiples testing corrected significance threshold *α*=0.05. **(A)** Hierarchical cluster analysis (HCL) shows clustering of samples according to sample type. Rows represent proteins. PQI values were *z*-score transformed. Proteins are colored according to their abundance. *denotes decrease in abundance under effect of only flg22 or nlp20. # denotes increase in abundance under effect of flg22 only. F: flg22 treated, N: nlp20 treated, C: control (untreated), and a: technical replicate. **(B)** STRING database binary protein interaction network of Prohibitins with Cytochrome C generated when indicated clusters were used as input sets. **(C)** STRING database interaction networks of ribosomal and associated proteins when indicated clusters were used as input sets. String search was done using experiments, databases, co-occurrence and co-expression as interaction sources and showing only interactions between proteins in the input set. Edges in the string interaction network indicate both functional and physical protein associations.

To expand on the putative set of proteins imported into the nucleus following elicitation of immunity we were interested in identifying quantitative changes in protein abundance in the nuclear proteome in the three biological scenarios (control, flg22, and nlp20). To this end we looked at PSM count used as protein quantification index (PQI) in all three biological replicate experiments and performed multiple sample significance testing (ANOVA), with FDR multiples testing corrected significance threshold *α*=0.05, followed by *post hoc* test. Ninety-three proteins showed a significant change in their abundance between conditions (Supplementary File 1- Table 13). Hierarchical clustering of these proteins showed that the three biological scenarios produced specific clusters (Figure 3A). The protein dendrogram was divided into four main clusters as follows: proteins with decreased abundance in the nucleus following either flg22 or nlp20 stimulus (cluster 1), proteins with increased abundance in the nucleus following nlp20 stimulus (cluster 2), proteins with increased abundance in the nucleus following nlp20 and flg22 stimulus (cluster 3), proteins with increased abundance in the nucleus following flg22 (cluster 4; Figure 3A; Supplementary File 1- Table 14). The four clusters comprise of proteins showing significant change in their abundance comparing elicited immunity to control. The proteins in these four clusters should play potential physiological roles in the nucleus during Pattern-triggered immunity (PTI). The 93 statistically significant proteins were annotated as being involved in several cellular processes such as mRNA processing, nucleotide binding, rRNA binding and include some protein families such as ribosomal proteins and prohibitin proteins.

The proteins whose abundance increased upon stimulus with an elicitor (proteins in clusters 2, 3, and 4) were input into the STRING protein interaction database to identify potential physical interactions between them and putatively infer function of proteins in complexes. The abundance of all of the members of the prohibitin family which are prohibitins 1, 2, 3, 4, and 6 increased significantly upon treatment with both flg22 and nlp20 (Supplementary File 1- Table 14) and all interacted physically with one another (Prohibitins connected by experimental evidence in the STRING interaction network). This prohibitin complex was expanded by three members of the mitochondrial bc_1_ complex, MPPBETA (AT3G02090) and Cytochrome C1 family proteins AT5G40810 and AT3G27240 (Figure 3B). The results indicate the possible function of these proteins together in the nucleus in PTI.

Nineteen ribosomal proteins showed a significant change in their abundance in between untreated and flg22 and nlp20 treated samples. Ten of these increased significantly in their abundance whereas the abundance of nine decreased significantly (Supplementary File 1- Table 14). The abundance of three ribosomal proteins (S4, S5, and L27e) was elevated upon either flg22 or nlp20 treatment. Conversely, the abundance of five ribosomal proteins (L13, L14p, L17, L24e, and L29) increased specifically upon exposure of the protoplasts to nlp20 and the abundance of two ribosomal proteins (L16p and S11) specifically upon exposure to flg22. Protein interaction analysis showed a core set of ribosomal proteins interacting in both PTI scenarios (Figure 3C, top left panel). This core cluster however differentially expanded as proteins responding only to flg22 or nlp20 were added to the common input set (Figure 3C, bottom left and right panels). This was particularly pronounced following elicitation of PTI by flg22 (Figure 3C, right panel). It has been reported by us (Bassal et al., 2020) that ribosome composition is promiscuous dependent on cellular state and our results imply the same in the context of ribosome assembly in the nucleus.

## Discussion

### Defining the Nuclear Proteome

In this study the *A. thaliana* nuclear proteome was investigated following treatment of protoplasts cultured in the absence of light with two elicitors of PTI, flg22 and nlp20. Despite the central role of the nucleus in regulating gene expression, the plant nuclear proteome remains somewhat understudied (Narula et al., 2013; Petrovská et al., 2015; Yin and Komatsu, 2016). An early study using two-dimensional gel-electrophoresis (2-DE) characterized the nuclear proteome upon cold stress but the coverage was limited (Bae et al., 2003). Several studies have reported nuclear sub-proteomes such as nucleolar proteins, nuclear matrix or chromatin associated proteins and nuclear envelope (Calikowski et al., 2003; Pendle et al., 2005; Sakamoto and Takagi, 2013; Bigeard et al., 2014; Chaki et al., 2015; Tang et al., 2020). Also the nuclear proteome has been studied in tomato, potato, rice and barley (Petrovska et al., 2014; Howden et al., 2017; Narula et al., 2019; Rajamaki et al., 2020). In barley the authors employed fluorescence assisted cell sorting (FACS) to purify nuclei from plant tissue. Only recently however three studies have described the *Arabidopsis* nuclear proteome comprehensively, employing LC–MS (Palm et al., 2016; Goto et al., 2019; Mair et al., 2019). Mair et al. (2019) additionally used *in vivo* protein proximity biotin labeling to affinity purify nuclear proteins with high specificity. Our study complements these three, achieving a similar amount of proteins and thus similar comprehensive coverage in the context of plant immunity.

Organelle proteomes, including the proteome of the nucleus, are by definition smaller than the total cellular proteome which comprises around 10,000 proteins at any given time (Nagaraj et al., 2011). Their qualitative composition, however, may be substantially more dynamic because of biological context dependent protein in- and export. The use of the plant protoplast to isolate the nuclei was successful and produced high quality nuclei in a round and intact form (Figure 1A). Here we sought to gain a first impression of these molecular trafficking processes on a large scale by use of the LC–MS technology. Retrograde signaling from the chloroplast to the nucleus in plant immunity is well known. In our work we used cells cultured in the dark without chloroplasts to avoid the inevitable co-purification of these organelles with nuclei. Thus, our study does not provide any insights into protein import from the chloroplast. The isolated nuclear fraction was of high purity and was enriched in histones and not in cytoplasmic or mitochondrial markers (Figures 1D,E), so our results disclose a number of candidate proteins that may be imported into the nucleus from these locations upon elicitation of PTI by both flg22 and nlp20.

An approach based on implementing a cut off limit was used to differentiate between nuclear and non-nuclear proteins. Generally cytoplasmic markers such as actin and phosphoenolpyruvate carboxylase are abundant in the cytoplasm and not expected to be found in the nucleus (Garcia et al., 2010; Genenncher et al., 2016; Dalmadi et al., 2019). Therefore, the cytoplasmic markers can act as representatives for the non-nuclear protein contaminants when found in the nucleus and their nuclear protein fraction (Npf_n_) can be used as cut off limit to curate the nuclear proteins identified in the nuclear fraction. Arguably the median Npf_n_ values of the cytoplasmic markers (used as a cut-off limit) and the mitochondrial markers were around 0.25, indicating a large portion of the proteins we considered as genuinely localized in the nucleus were more abundant in the cellular fraction. Nonetheless, we think our approach is valid, because the selected markers are explicitly not nuclear and a substantial portion of the nuclear proteome is not facultative, i.e., found in other compartments and potentially underlying some trafficking. Indeed, the purity of the curated nuclear proteins was verified and 89% were either experimentally verified nuclear proteins or proteins annotated/predicted to be localized in the nucleus. This reveals the success of the procedure and experimental approach used in characterizing the nuclear proteome.

Proteins identified and annotated to the nucleus were involved in diverse nuclear functions. Transcription factors and transcriptional regulators control gene activity. Ribosomal proteins are part of the ribosomal biogenesis process (Watkins and Bohnsack, 2012; Woolford and Baserga, 2013; Turowski and Tollervey, 2015). Nucleotide binding proteins (DNA or RNA binding), modulate gene expression and kinases are components of signal transduction cascades that regulate gene expression (Hucho and Buchner, 1997). Translation initiation factors act as regulatory players in the translation process. WD40 proteins participate in various biological regulatory processes such as histone modifications, histone recognition and transcriptional regulation (Znaidi et al., 2004; Suganuma et al., 2008).

### Dual Targeting of Proteins

Proteins exert their functions in one or more organelles and it has been recognized that dual targeting of proteins in various developmental or stress response scenarios is an important but still understudied phenomenon especially in plants. Dual targeting was first identified 1995 (Creissen et al., 1995) but research in the last 20years suggests that it may be a widespread event leading to the diversification of protein function (Krause and Krupinska, 2009; Sharma et al., 2018; Krupinska et al., 2020). Query of the SUBA4 database with our list of nuclear proteins identified several hundred proteins with known alternative sub-cellular localization possibly implicating them as dual targeted. Protein dual targeting has been most studied using FP based approaches and our list presents candidates that could be investigated further for confirmation.

### Nuclear Proteins Import Under flg22 and nlp20 Stimuli

In this work the nuclear import was investigated under flg22 and nlp20 as triggers of immune response. Plant immunity is a complex process that involves an array of protein and small molecule, particularly phytohormone, signaling, large scale reprograming of transcription and ultimately proteome remodeling. Our previous study (Bassal et al., 2020) has shown that 16h after flg22 treatment PTI is fully induced and proteome remodeling is complete as opposed to shorter time points of 1 and 3h when transcriptional processes are more predominant and proteins accumulate or are depleted. For this reason, we again chose a long exposure time of 16h to ensure full penetrance of PTI to the proteome. This time point will capture both early and late processes including import of differentially expressed proteins into the nucleus because of continuous exposure to the PAMP and full elicitation of immunity over a prolonged period. A limitation is that we cannot differentiate between proteins present before elicitation and proteins synthesized in the course of PTI. We believe this is valid because many proteins that are imported into the nucleus such as TFs that drive flg22 induced gene expression themselves underlie PAMP induction.

Two lists of proteins were identified as potential candidates for import under both treatment conditions (157 proteins for flg22 condition and 73 proteins for nlp20 condition). Generally, protein transport into the nucleus is controlled by different mechanisms. Proteins smaller than 40–60kDa are diffused in a passive manner but larger proteins need to be recognized by the nuclear transport receptors which bind the nuclear localization signals (NLS) on those proteins and facilitate import (Weis, 2003; Wang and Brattain, 2007; Mohr et al., 2009; Chook and Suel, 2011; Grossman et al., 2012; Tamura and Hara-Nishimura, 2014; Christie et al., 2016; Timney et al., 2016). In addition, alternative mechanisms were also investigated (Guinez et al., 2005; Imamoto and Kose, 2012). In the flg22 list 33% of the proteins were predicted to have NLS, the molecular weight of the rest of the proteins in flg22 list were checked and 71% of them were less than 40–60kDa. Similarly, in the nlp20 list 33% of the proteins were predicted to have NLS and 88% of the rest of the proteins were less than 40–60kDa. Therefore, the proteins we postulate to be imported fulfill the requirements for the transport into the nucleus.

Proteins function in a specific manner regarding time and location. Therefore, the designated proteins needed to be activated and recruited to certain subcellular locations only when required (Wiatrowski and Carlson, 2003; Michaelson et al., 2008). The activation could be through a signal transduction cascade that activates the protein by post translational modifications (Di Ventura and Kuhlman, 2016). In addition, nucleoporins regulate selectively the passage of certain stress-sensible proteins (Yang et al., 2017) by undergoing conformational changes upon receptor activation and allowing transport of specific macromolecules (Gu et al., 2016). In this work the nuclear import was investigated under flg22 and nlp20 stimulus, implying that the flg22 and nlp20 induced responses could directly or indirectly regulate nuclear import of the selected two sets of proteins by one of the above mentioned mechanisms.

Most mitochondrial proteins contain transit peptides in their primary structure. Transit peptides are usually removed by mitochondrial processing peptidases (MPP) following import into mitochondria (Hawlitschek et al., 1988). Fifty-one kilodalton subunit of complex I and NAD-dependent malic enzyme 2 are mitochondrial proteins that were previously reported also in the nucleus (Iglesias et al., 2013; Palm et al., 2016). Fifty-one kilodalton subunit of complex I was identified as a substrate for the mitochondrial localized peptidase ICP55, which is a secondary processing peptidase that removes phenylalanine (F) from its MPP-processed form (Carrie et al., 2015). Phenylalanine was the first amino acid preceding the non-tryptic N-terminal peptide we identified in our MS results by way of no enzyme specificity search (Table 2). This may explain the non-tryptic cleavage site, giving a hint of possible of primary processing of the protein’s transit peptide by MPP followed by secondary removal of phenylalanine in the mitochondria before trafficking to the nucleus. Interestingly, phenylalanine was again the first amino acid before the identified non-tryptic peptide for the NAD-dependent malic enzyme 2. In addition, ICP55 has a general consensus motif of RX (F/Y/I/L) (S/A) (S/T) where it removes the amino acids F, Y, I, or L (Carrie et al., 2015). As shown in Table 2, the NAD-dependent malic enzyme 2 contains the ICP55 processing motif RC (F) (S) (T). This suggest that NAD-dependent malic enzyme 2 could also be processed by ICP55 after removal of the transit peptide by MPP. These MS findings require further verification by orthogonal methods.

### Comparison of Nuclear Proteomes Under flg22 and nlp20 Stimuli

The nuclear proteomes were investigated for quantitative changes in protein abundance in the three biological scenarios: untreated control, flg22 and nlp20 treatment. Ninety-three proteins showed statistically significant changes in their abundance upon elicitation of one or both forms of PTI when compared to control. We will focus in the discussion on two proteins families: ribosomal proteins and prohibitin proteins.

### Ribosomal Proteins

The ribosomes are the cellular machinery required for the process of protein synthesis. The maturation of the ribosomes is required for its function, the process of ribosome maturation is called ribosome biogenesis. Ribosome biogenesis involves association of ribosomal proteins with rRNA to constitute the ribosomal subunits (Thomson et al., 2013; Sáez-Vásquez and Delseny, 2019). Primary steps of ribosome biogenesis exist in the nucleus before exportation to the cytoplasm (Brown and Shaw, 1998; Woolford and Baserga, 2013; Stępiński, 2014; Henras et al., 2015). In addition to their function in ribosome biogenesis and protein synthesis ribosomal proteins have various extra-ribosomal functions (Zhou et al., 2015), for example, transcription regulation and histone binding in the nucleus (Denmat et al., 1994; Tchórzewski et al., 1999; Ni et al., 2006; Dieci et al., 2009; Tu et al., 2011). Therefore, ribosomal proteins have been identified in the nucleus in many studies for instance in *A. thaliana* (Pendle et al., 2005; Chaki et al., 2015; Palm et al., 2016). In this work ribosomal proteins were also identified in our nuclear proteome, 19 of them had a significant change in their abundance between control and elicited immunity. Interestingly, these ribosomal proteins showed different abundance in the nucleus when treated with the two elicitors flg22 and nlp20 as mentioned in the results. These elicitor specific changes in abundance suggest that ribosomal proteins do not act similarly and have different functions in the two types of PTI. Accordingly, we can speculate that the ribosomal proteins with increased abundance in the nucleus under flg22 and nlp20 compared to control (10 proteins: five proteins specific to nlp20, two protein specific to flg22 and three proteins for both flg22/nlp20) play an active role in the nucleus during the immune response under different stimuli in *Arabidopsis*. On the other hand, the ribosomal proteins with decreased abundance in the nucleus under flg22 and nlp20 compared to control (nine proteins) have a repressed function in the nucleus during the *Arabidopsis* immune response. In a previous study the ribosomal protein transcripts were investigated in *Vanilla planifolia* when infected with *Fusarium oxysporum* (Solano de la Cruz et al., 2019). Seven ribosomal proteins that showed an increase in abundance in the nucleus after elicited immunity in our study also showed an increase in their transcript expression patterns in *Vanilla* after 2days of *Fusarium* infection. These seven protein families are: ribosomal protein L14p/L23e family protein, ribosomal L29 family protein, ribosomal protein L13 family protein, ribosomal protein L24e family protein, ribosomal protein L17 family protein, ribosomal protein S4 (RPS4A) family protein and ribosomal protein S5 domain two-like superfamily protein. In addition, ribosomal protein L24e family protein was also detected exclusively in the nucleus of the *cerk1* background in *Arabidopsis* after chitosan treatment (triggering a MAMP-like response). The authors also observed that the ribosomal proteins were overrepresented after chitosan treatment (Fakih et al., 2016). This suggest that these seven ribosomal proteins have distinct functions in plant immunity in different plants elicited by different pathogens and promiscuity of ribosomal proteins in ribosome assembly is known. This functional promiscuity is reflected by the different protein interactions undergone by the ribosomal proteins in the two PTI scenarios (Figure 3C).

### Prohibitins

Prohibitins are group of conserved proteins in eukaryotes including plants (Van Aken et al., 2010). They were reported to have several functions as scaffold proteins in mitochondrial biogenesis and immunity (Yu, 2019). Prohibitins participate in plant defense response and in protection against stress, for example: they are involved in the rice defense response against fungi (Takahashi et al., 1999, 2003). PHB1 and PHB2 are localized in the mitochondria and participate in its biogenesis and in the plant response to stress in *Nicotiana benthamiana* (Ahn et al., 2006) and PHB3 is additionally localized to the chloroplast where it regulates the production of salicylic acid under UV and biotic stress in *Arabidopsis* (Seguel et al., 2018). Besides their localization in the mitochondria and chloroplasts, prohibitins have also been reported in the nucleus and act as transcription regulators in eukaryotes (Mishra et al., 2006; Thuaud et al., 2013; Peng et al., 2015; Huang et al., 2019). In addition, PHB3 were localized by FP in the nucleus in *A. thaliana* (Pendle et al., 2005; Christians and Larsen, 2007; Huang et al., 2019) and possible shuttling between mitochondria and nucleus were also suggested (Yu, 2019). In *A. thaliana* five prohibitins are expressed (PHB1, PHB2, PHB3, PHB4, and PHB6; Van Aken et al., 2007) and all of them were identified in our nuclear proteome with increased abundance following treatment with both flg22 and nlp20. This indicates that the prohibitin family plays a role in the *Arabidopsis* defense response in the nucleus. In previous studies, PHB2 was detected exclusively in the nucleus of *cerk1 Arabidopsis* plant after chitosan treatment (triggering a MAMP-like response; Fakih et al., 2016) and prohibitin protein was also identified in the nucleus of *Solanum lycopersicum* with increased abundance after 24h infection with *Phytophthora capsici* compared to non-infected plants (Howden et al., 2017). The results of these two studies support our findings of a probable role of prohibitins in the nucleus during the plant immune response. In addition, all five prohibitins interacted with each other and Cytochrome C (Figure 3B), another mitochondrial protein whose abundance also increased in the nucleus in PTI. Cytochrome C has been shown to have functions in the nucleus such as DNA damage repair and interaction with histone proteins (Gonzalez-Arzola et al., 2019) in addition to its well-known function in the mitochondrial respiratory chain. It is therefore tempting to speculate, that the prohibitins may act as a scaffold to traffic Cytochrome C from the mitochondrion to the nucleus in PTI.

## Data Availability Statement

All raw and metadata have been deposited to ProteomeXchange Consortium via the Pride partner repository with the dataset identifier PXD024349 and can be found at: http://www.proteomexchange.org.

## Author Contributions

WH conceived and oversaw the study. MAy, MAb, and WH designed the experiments. MAy, MAb, MH, CP, and DT performed the experiments. MAy, MH, MS, and WH analyzed the data. MAy and WH wrote the manuscript. All authors contributed to the article and approved the submitted version.

## Funding

MAy is funded by DFG 4006811449/GRK2498. MAb is funded by DFG grant HO 5063/2-1. This work was also supported by de.NBI (FKZ 031 A 534A), a project of the BMBF (BundesministeriumfürBildung und Forschung).

## Conflict of Interest

The authors declare that the research was conducted in the absence of any commercial or financial relationships that could be construed as a potential conflict of interest.

## Publisher’s Note

All claims expressed in this article are solely those of the authors and do not necessarily represent those of their affiliated organizations, or those of the publisher, the editors and the reviewers. Any product that may be evaluated in this article, or claim that may be made by its manufacturer, is not guaranteed or endorsed by the publisher.

## Supplementary Material

Supplementary File 1 Tables containing the protein lists from the study.

Supplementary File 2 Tables containing detailed (unfiltered) proteome discoverer exported results.

Supplementary Figure 1 Non-cropped western blot of nuclear and cellular proteins with anti-Histone H3 antibody. Two independent experiments are shown, N denotes nuclear and C cellular.
