# Supplementary figures and images for: LC–MS Based Draft Map of the Arabidopsis thaliana Nuclear Proteome and Protein Import in Pattern Triggered Immunity
Source: Front Plant Sci. 2021 Nov 8;12:744103. doi: 10.3389/fpls.2021.744103 (PMC8630587; doi:10.3389/fpls.2021.744103)

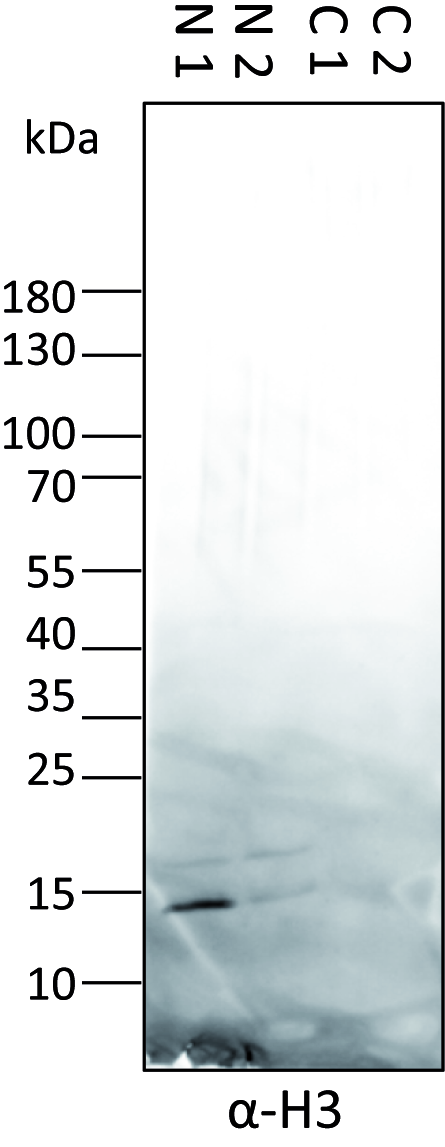

Supplement: Supplementary Figure 1 — Non-cropped western blot of nuclear and cellular proteins with anti-Histone H3 antibody. Two independent experiments are shown, N denotes nuclear and C cellular. [file Image_1.TIF]
